# Comparison between ultrasound and noncontrast helical computed tomography for identification of acute ureterolithiasis in a teaching hospital setting

**DOI:** 10.1590/S1516-31802007000200007

**Published:** 2007-03-04

**Authors:** Luís Ronan Marquez Ferreira de Souza, Suzan Menasce Goldman, Salomão Faintuch, Juliano Ferreira Faria, Daniel Bekhor, Dario Ariel Tiferes, Valdemar Ortiz, Peter Choyke, Jacob Szejnfeld

**Keywords:** Spiral computed tomography, Ultrasonography, Ureteral calculi, Lithiasis, Flank pain, Tomografia computadorizada espiral, Ultra-sonografia, Cálculos ureterais, Litíase, Dor no flanco

## Abstract

**CONTEXT AND OBJECTIVE:**

Recent studies have shown noncontrast computed tomography (NCT) to be more effective than ultrasound (US) for imaging acute ureterolithiasis. However, to our knowledge, there are few studies directly comparing these techniques in an emergency teaching hospital setting. The objectives of this study were to compare the diagnostic accuracy of US and NCT performed by senior radiology residents for diagnosing acute ureterolithiasis; and to assess interobserver agreement on tomography interpretations by residents and experienced abdominal radiologists.

**DESIGN AND SETTING:**

Prospective study of 52 consecutive patients, who underwent both US and NCT within an interval of eight hours, at Hospital São Paulo.

**METHODS:**

US scans were performed by senior residents and read by experienced radiologists. NCT scan images were read by senior residents, and subsequently by three abdominal radiologists. The interobserver variability was assessed using the kappa statistic.

**RESULTS:**

Ureteral calculi were found in 40 out of 52 patients (77%). US presented sensitivity of 22% and specificity of 100%. When collecting system dilatation was associated, US demonstrated 73% sensitivity, 82% specificity. The interobserver agreement in NCT analysis was very high with regard to identification of calculi, collecting system dilatation and stranding of perinephric fat.

**CONCLUSIONS:**

US has limited value for identifying ureteral calculi in comparison with NCT, even when collecting system dilatation is present. Residents and abdominal radiologists demonstrated excellent agreement rates for ureteral calculi, identification of collecting system dilatation and stranding of perinephric fat on NCT.

## RESUMO

**CONTEXTO E OBJETIVO:**

Estudos atuais demonstram que a tomografia computadorizada helicoidal sem contraste (TC) apresenta maior acurácia do que a ultra-sonografia (US) no diagnóstico da ureterolitíase aguda, porém, poucos estudos a esse respeito foram realizados em atendimento radiológico de urgência de hospital universitário. Nossos objetivos foram comparar a sensibilidade diagnóstica da US com a TC realizadas por residentes no diagnóstico de ureterolitíase aguda e comparar a análise da TC interpretada por residentes e radiologistas experientes.

**TIPO DE ESTUDO E LOCAL:**

Estudo prospectivo de 52 pacientes com cólica renal aguda, que foram submetidos a exame de US seguido de TC em período máximo de oito horas no Hospital São Paulo.

**MÉTODOS:**

Os exames de US foram realizados por médicos residentes e conferidos pelos preceptores, já os de TC foram analisados por outro residente e posteriormente analisados por três radiologistas independentes.

**RESULTADOS:**

Nos 52 pacientes analisados foram encontrados 40 cálculos ureterais na TC (77%). A US apresentou uma sensibilidade de 22% e especificidade de 100%, que aumentou para 73% e 82% respectivamente, quando se associou a identificação da dilatação do sistema coletor. A TC analisada pelo residente e pelos radiologistas apresentou uma excelente correlação para identificação do cálculo ureteral, para heterogeneidade da gordura peri-renal e para dilatação do sistema coletor.

**CONCLUSÕES:**

A US realizada pelos residentes tem menor sensibilidade no diagnóstico da litíase ureteral, quando comparada à TC, mesmo quando associada à presença de dilatação do sistema coletor. Residentes e radiologistas especialistas apresentaram excelente concordância no diagnóstico de litíase ureteral.

## INTRODUCTION

Since its first introduction by Smith et al. in 1995,^1^ noncontrast helical computed tomography (NCT) has evolved into a tool for rapid examination of patients suspected of having ureterolithiasis, without the limitations of plain films, intravenous urography and ultrasound.^2-4^ NCT has become the method of choice for evaluating patients with acute renal colic.^5-8^

Transabdominal ultrasound (US) has the advantages of being universally available, not exposing the patient to radiation and being independent of kidney function.^9-10^ Because of these advantages, US is preferred by referring clinicians for evaluating acute renal colic.

Recent studies^9-11^ have shown NCT to be more effective than US for imaging ureterolithiasis in patients with acute renal colic. However, to our knowledge, there are few studies directly comparing these techniques in an emergency teaching hospital setting.^12^

## OBJECTIVE

The twofold purpose of our study was to compare the diagnostic sensitivity of US and NCT performed by radiology residents for diagnosing ureterolithiasis, in patients with acute renal colic; and to assess interobserver agreement regarding NCT interpretation by a group of senior residents and experienced radiologists.

## METHODS

Between February and July 2002, we conducted a prospective study on 52 consecutive patients referred from our emergency department for evaluation of acute renal colic. Renal colic was defined as a painful symptom relating to possible obstruction of the collecting system that started as an acute flank pain and which made the patient seek medical help.

The study protocol had previously been approved by our institutional ethics committee and all patients gave their consent for their participation.

The patients underwent both US and NCT within eight hours of the onset of colic. The exclusion criteria were other known renal diseases or imaging signs of pyelonephritis, chronic renal insufficiency, nephrocalcinosis and staghorn calculus.

The US examination was performed transabdominally, after ingestion of water. Sonography was performed by senior radio-logy residents and immediately checked by attending radiologists, using a Philips SD800 scanner (Philips Medical Systems, Eindhoven, Netherlands) with a convex (curved phased array) transducer (2-5 MHz) and transducer frequencies selected to optimize the imaging of the kidneys, ureters and bladder. The US diagnosis of ureteral calculi required the demonstration of an intraluminal hyperechoic structure causing acoustic shadowing. The presence of collecting system dilatation was also evaluated. No patient underwent transvaginal or transrectal sonographic examination.

NCT scans were acquired after US examination, on a Tomoscan EV-EV1 (Philips Medical Systems, Eindhoven, Netherlands) using Secura Release 1.3 software. The scan parameters included helical data acquisition, with section thickness 3-5 mm, using 120 kV and 200 mAs and a pitch of 1-1.5. Images were obtained during apnea, from the top of the kidneys to the base of the bladder, and no contrast medium was used. The NCT images were interpreted by a senior resident, using an electronic workstation (Philips), and subsequently reviewed by three experienced abdominal radiologists in a blinded manner.

The NCT scan analysis included identification and localization of ureteral calculi, and evaluation for the presence of the following signs: intrarenal collecting system and/or ureteral dilatation, and stranding of perinephric and periureteral fat. Incidental diagnoses were also recorded.

Once the three experienced observers had completed their independent reviews for the interobserver investigation, all the cases in which there were disagreements about the presence of collecting system obstruction or ureteral calculi on NCT scans were reevaluated. Any differences were resolved by consensus.

The locations of the calculi were defined as proximal (above the sacroiliac joints, SIJ), mid (overlying the SIJ), distal ureteral (below the SIJ), or at the ureterovesical junction (UVJ). Stone size was measured at the maximum diameter within the plane of the axial CT section; the measurement was made perpendicularly to the course of the ureter, on a workstation. Stones were considered to be definitively present when recovered in urine, extracted during urological procedures or clearly shown by CT interpretation.^13^

Differences in sensitivity and specificity were calculated using the McNemar test. Interobserver variability for the detection of ureteral calculi on US and CT scans was evaluated using the kappa statistic.^14^ A p-value of less than 0.05 was taken to indicate a statistically significant difference.

## RESULTS

**Figure 1:**
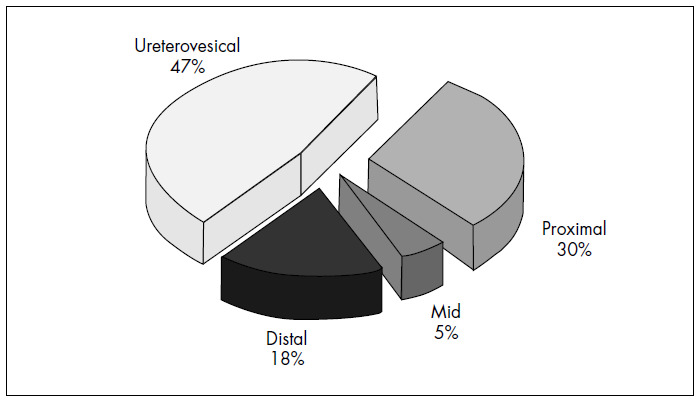
Localization of the 40 ureteral stones founded on noncontrast helical computed tomography of 52 patients.

Among the 52 patients studied, 40 ureteral stones were detected on NCT, thus giving a prevalence of 77%. The locations of the calculi were: UVJ (47%), proximal (30%), distal (18%) and mid-ureteral (5%) (Figure 1). The mean calculus size (longest axis) was 5 mm, with a range from 2 mm to 14 mm. No patient had more than one stone. In all cases, both exams were performed within eight hours, with an average of four hours between US and NCT.

Among the 12 patients who did not have ureteral stones identified by CT, one had infected renal cysts and four were considered to have had spontaneous passage of ureteral calculi, because unilateral collecting system dilatation was found on the symptomatic side, without other image findings. For the remaining seven patients, no definitive diagnosis was possible.

As shown in Table 1, CT read by abdominal radiologists identified 40 calculi, and US demonstrated only nine, thus corresponding to sensitivity of 22%, specificity of 100% and accuracy of 40%. The agreement between the US performed by the group of senior residents and the NCT read by the experienced radiologists was very low (k = 0.06). In all cases when CT was negative for ureteral stones, the results matched those from US.

**Table 1:** Comparison between ultrasound and noncontrast helical computed tomography for identifying ureteral stone

	NCT positive for ureteral stone	NCT negative for ureteral stone	Total
US positive for ureteral stone	9	0	9
US negative for ureteral stone	31	12	43
**Total**	**40**	**12**	**52**

NCT = noncontrast helical computed tomography; US = ultrasound; k = 0.06 (6%) for agreement between senior residents and experienced radiologists.

When unilateral intrarenal collecting system dilatation was used as an indirect sign of ureteral calculi (Table 2), the sensitivity of US improved to 73%, but the specificity decreased to 82%, with an overall accuracy of 75%. The agreement between US including collecting system dilatation, read by senior residents, and NCT read by the experienced radiologists improved to a moderate level (k = 0.42).

**Table 2:** Comparison between ultrasound and noncontrast helical computed tomography for identifying ureteral stone and/or intrarenal collecting system dilatation

	NCT positive for ureteral stone and/or intrarenal collecting system dilatation	NCT negative for ureteral stone and/or intrarenal collecting system dilatation	Total
US positive for ureteral stone and/or intrarenal collecting system dilatation	30	2	32
US negative for ureteral stone and/or intrarenal collecting system dilatation	11	9	20
**Total**	**41**	**11**	**52**

NCT = noncontrast helical computed tomography; US = ultrasound; k = 0.42 (42%) for agreement between senior residents and experienced radiologists.

To evaluate the interobserver agreement between residents and experienced abdominal radiologists, we used the kappa statistic and the results are demonstrated in Table 3. The agreement was high for identification of calculi (k = 0.81), collecting system dilatation (k = 0.75) and stranding of perinephric fat (k = 0.78), but moderate for stranding of ureteral fat (k = 0.41) and for ureteral dilatation (k = 0.46).

**Table 3:** Interobserver agreement in computed tomography evaluation (residents versus experienced radiologists)

	Residents	Experienced radiologists	Kappa
Identification of ureteral stone	36	40	0.81
Intrarenal collecting system dilatation	29	36	0.75
Perinephric fat stranding	10	13	0.78
Ureteral dilatation	16	31	0.46
Ureteral fat stranding	3	10	0.41

## DISCUSSION

Ultrasound has many desirable features as an imaging method. It is inexpensive, does not expose the patient to ionizing radiation and can be performed at the patient’s bedside. Unfortunately, the sensitivity of US is highly variable for evaluating patients with acute renal colic and depends on stone size, examiner experience and patient conditions. Fowler et al.^15^ found that US is a poor means for demonstrating stones smaller than 4.0 mm. One of the main disadvantages of US is that the identification of a stone within the ureter is frequently hindered by the patient’s body habitus or by obscuring of portions of the ureter due to overlying bowel gas.^7,16^

Training for performing basic US examinations is a quick process for radiology residents. However, a higher level of skill, knowledge and familiarity with US findings is needed when the stones are not at the ureteral junctions, or whenever alternative diagnoses must be made.^9^ Ultrasound is very sensitive in depicting the anatomical changes associated with obstruction of the collecting system.^17^ Nonetheless, we found that the sensitivity of US was only 22% in comparison with NCT, which is inadequate for routine use.

The causes of false negative US examinations include minor dilatation of the collecting system during early obstruction, forniceal rupture, extrarenal pelvis and decreased renal output.^8^

**Figure 2:**
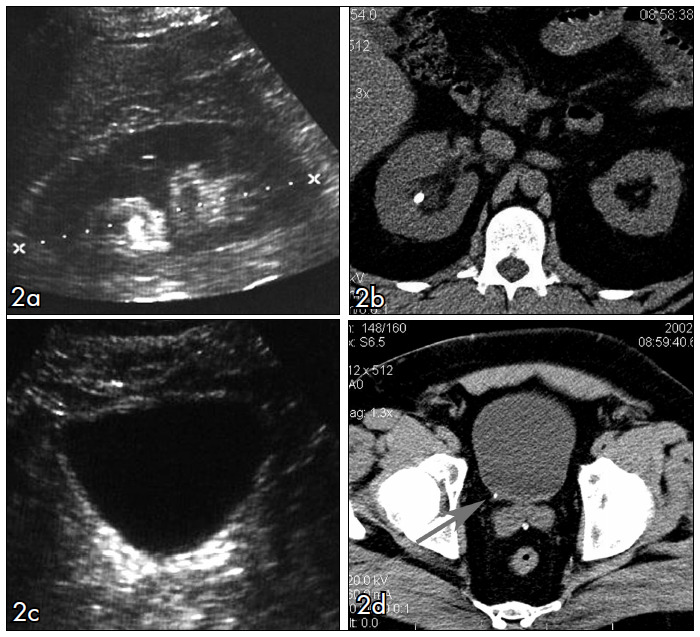
33-year old man with right-sided renal colic. Noncontrast helical computed tomography (CT) and abdominal ultrasound (US) in the same patient. (a) Coronal identification of the right kidney (between calipers) with a renal stone and mild intrarenal collecting system dilatation. The same findings were found on CT scan (b). In the US scan of the bladder and right ureterovesical junction (c), the radiology resident did not find the small stone (3 mm), shown in CT (d).

**Figure 3:**
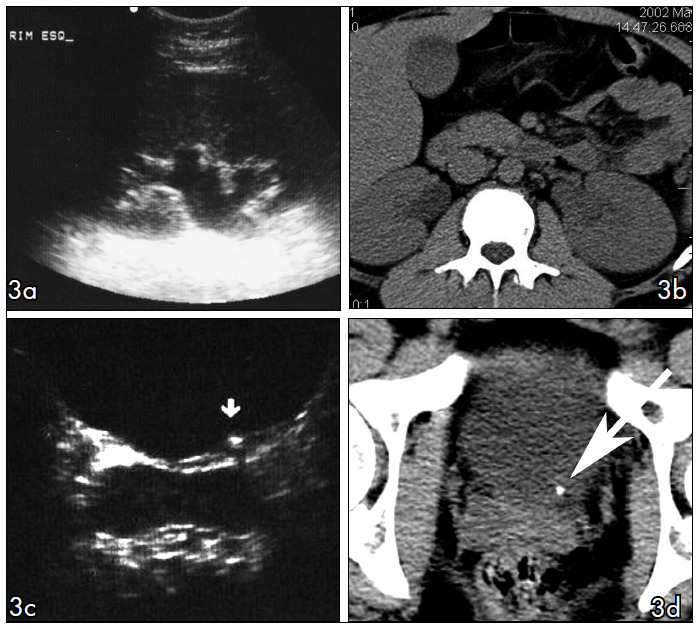
Comparison between ultrasound (US) and computed tomography (NCT) in two different patients. (a) Coronal identification of the left kidney showing collecting system dilatation, which was confirmed in NCT (b). US scan at bladder level showing stone at left ureterovesical junction (arrow in c), confirmed by NCT (d).

In our study, US performed by the group of senior residents presented limited accuracy in comparison with CT read by the experienced observers, for identifying ureteral calculi (Figures 2 and 3). The accuracy of US improved from 40% to 75% with the association of unilateral collecting system dilatation as a secondary sign for ureteral calculi. However, the specificity decreased because there were more frequent false positive examinations.

All stones, regardless of their chemical composition, are generally depicted by NCT. It has recently been reported that stones formed as concretions of crystals of protease inhibitor (e.g. indinavir), which are relatively radiolucent, are the only calculi that are undetectable on CT.^18-20^

Unenhanced helical CT is an extremely fast and efficient imaging method. In our institution, the time taken to perform a CT scan was determined to be approximately seven minutes (room time). The test is not affected by the presence of increased amounts of bowel gas or by obesity.^20,21^ Thus, unenhanced helical CT is currently the imaging test of choice for evaluating patients with acute flank pain for whom the clinical diagnosis is uncertain.^22-29^

In the CT evaluation of ureteral calculi, the agreement between the group of senior residents and the consensus among the abdominal radiologists were very good, as shown in Table 3. The interobserver agreement for identifying the ureteral stone was almost perfect (k = 0.81). The findings in our study are concordant with Freed et al.,^12^ who found a very good interobserver agreement in evaluations of ureteral stone disease, comparing experienced radiologists with radiology residents (k = 0.65-0.67). To our knowledge, this was the only previous study with such analysis undertaken within a teaching hospital setting.

**Figure 4:**
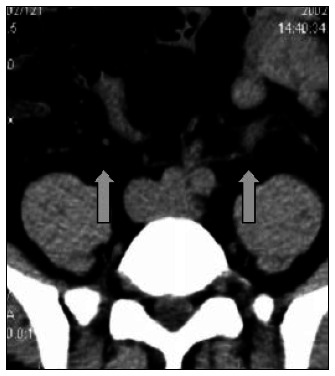
Collecting system dilatation. Noncontrast helical computed tomography (NCT) on a patient with a stone in the distal left ureter, showing ureteral dilatation in comparison with the contralateral normal side (arrows).

**Figure 5:**
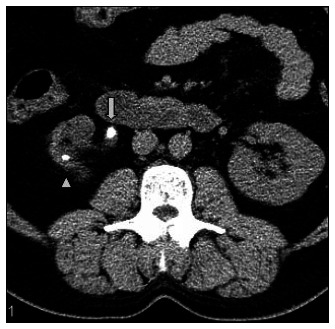
Ureteral fat stranding. Axial slice in noncontrast helical computed tomography (NCT). Stone identified in the proximal right ureter (arrow), associated with ureteral wall edema (tissue rim sign) and perinephric fat stranding. A stone in the kidney is also identified (arrow head).

The two most common secondary signs of ureteral obstruction observed in our study are also the two most commonly reported in the literature.^30-31^ These were intrarenal collecting system dilatation (74% of the cases) and ureteral dilatation (in 71% of the cases) (Figures 4 and 5).

When we analyzed the interobserver agreement for identifying intrarenal collecting system dilatation (k = 0.75) and stranding of perinephric fat (k = 0.78), we found substantial agreement between the residents and the experienced physicians. When we evaluated ureteral dilatation (k = 0.46) and stranding of ureteral fat (0.41), the interobserver agreement was only moderate. This may have been due to inexperience with what constitutes ureteral dilatation and the more subjective nature of periureteric fat stranding, compared with perinephric stranding.

One advantage of our study was the short time interval between the US and CT examinations, with an average interval of four hours. This was a limitation in previous studies, in which CT and US were obtained with longer intervals between them. Shorter intervals minimize the likelihood that the stone could have passed through prior to the second examination.^15^

Varanelli et al.^32^ investigated the relationship between duration of the flank pain and frequency of secondary signs of ureteral obstruction on unenhanced helical CT. Their study demonstrated that all the secondary signs, except nephromegaly, showed significantly increased frequency as the duration of flank pain increased. In our data, there was no statistical significance between the duration of pain and the frequency of identification of secondary signs.

The limitations of our study include the fact that we did not use unenhanced plain radiography, resistive index values or color Doppler evaluation of ureteral jets to increase the accuracy of our ultrasound examinations.^7,11,33,34^ The relatively low prevalence of non-urinary diagnoses in our patient population may have resulted from more accurate patient screening by the referring emergency department physicians.^11^

## CONCLUSIONS

Different methods are available to radiologists for evaluating patients with acute renal colic, but noncontrast helical CT has overwhelmingly become the diagnostic method of choice.^35,36^ The findings from this study have confirmed this within a teaching hospital setting, and have also demonstrated that the learning curve for NCT is faster, by showing that the interobserver agreement between experienced abdominal radiologists and senior residents is excellent for identifying ureteral calculi, intrarenal collecting system dilatation and perinephric fat stranding.
